# Deep Learning using Convolutional LSTM estimates Biological Age from Physical Activity

**DOI:** 10.1038/s41598-019-46850-0

**Published:** 2019-08-06

**Authors:** Syed Ashiqur Rahman, Donald A. Adjeroh

**Affiliations:** Lane Department of Computer Science & Electrical Engineering, West Virginia University, Morgantown, USA

**Keywords:** Biomarkers, Biomedical engineering, Epidemiology

## Abstract

Human age estimation is an important and difficult challenge. Different biomarkers and numerous approaches have been studied for biological age estimation, each with its advantages and limitations. In this work, we investigate whether physical activity can be exploited for biological age estimation for adult humans. We introduce an approach based on deep convolutional long short term memory (ConvLSTM) to predict biological age, using human physical activity as recorded by a wearable device. We also demonstrate five deep biological age estimation models including the proposed approach and compare their performance on the NHANES physical activity dataset. Results on mortality hazard analysis using both the Cox proportional hazard model and Kaplan-Meier curves each show that the proposed method for estimating biological age outperforms other state-of-the-art approaches. This work has significant implications in combining wearable sensors and deep learning techniques for improved health monitoring, for instance, in a mobile health environment. Mobile health (mHealth) applications provide patients, caregivers, and administrators continuous information about a patient, even outside the hospital.

## Introduction

A recent popular study^1^ showed that more than 27.5% of adults had insufficient physical activity worldwide. The study included 358 population-based surveys in 168 countries with a total of 1.9 million participants^2^. Numerous health risks such as hypertension, diabetes, mental health, and weight-gain are related directly to physical activity^1^. With aging, intensity of physical activity tends to decrease for older people^3,4^, and this is more evident for females^5^. Various organizations^2,6^ have recommended levels of physical activity for different age groups. However, the exact relationship between physical activity and aging is still unclear^7^. For instance, there is still the question of whether physical activity can predict age^8^.

The process of aging is complex and affects all biological systems. Age has a deep connection with health and mortality^9–11^. In general, a younger person is expected to have a better health condition, to be physically more active, and to have lower mortality hazard in comparison with a relatively older person. But two different people of the same chronological age may have very different health profiles and mortality hazards. This brings up an important classification of age, namely, chronological age versus biological age. Chronological age is based on the date of birth. However, biological age is a conceptual idea that a person’s true age can be different from his/her chronological age. Although biological age is a loosely used concept and lacks precise definition, it is often viewed as the true age of an individual^12^. Thus, biological age provides a better measure of the life expectancy of an individual than his or her chronological age. The common idea is to calculate biological age based on some age-dependent variables^13–16^, where chronological age may or may not be a required variable depending on the application. In this work, our focus is on biological age. In particular, we investigate the question of whether human physical locomotor activity as recorded using a wearable device can be used for reliable estimation of biological age in adults.

Klemera and Doubal method^12^ is the most popular approach to biological age prediction. The biological age (BA) estimates are derived based on minimizing the distance between biomarker points and defined regression lines, and the estimates are performed in a multi-dimensional space of all the biomarkers. They also used chronological age as an input parameter to predict BA. Liu *et al*.^17^ introduced the notion of “Phenotypic Age” based on a linear combination of age and clinical biomedical measures. These measures were selected using Cox proportional elastic net. Authors suggested that the proportional risk model can be used both for testing biological age performance, and for building a biological age model. Other approaches proposed include multiple linear regression (MLR)^18,19^. Principal component analysis have also been used to select features and then MLR was applied using the principal components^20^. Levine^9^ compared the performance of five BA estimation algorithms, and identified the Klemera and Doubal (KD) method as the most reliable predictor for mortality. The performance using BA was significantly better in comparison with using chronological age. Cho *et al*.^15^ studied various BA estimation methods to examine the relation with work ability index (WAI). WAI is a measure that reflects present health condition rather than how it changes over age. The KD method on PCA features produced relatively reliable results. Mitnitski *et al*.^11^ compared performance of frailty index (FI) with biomarker-based measures of BA. They employed the KD algorithm in predicting mortality. Belsky *et al*.^10^ described biological age as a reflection of ongoing longitudinal change within a person. They calculated study member’s BA at age 38 using the Klemera-Doubal method and parameters estimated from NHANES-III dataset. The study also tested the hypothesis that young adults with older biological age at age 38 were aging faster than young adults at the same chronological age, but with younger biological age. To quantify the pace of aging, longitudinal repeated measures are needed that track change over time. They analyzed within-individual longitudinal change in 18 biomarkers from the Dunedin Study across chronological ages 26 y, 32 y, 38 y to quantify each study member’s personal rate of physiological deterioration. In a more recent work, Belsky *et al*.^21^ compared different methods of BA estimation, including genomic, epigenetic, and blood biomarker measures.

Putin *et al*.^22^ studied the use of biomarkers in a deep learning framework for chronological age prediction. They utilized an ensemble of multiple deep neural networks (DNNs) trained on blood biomarkers. They employed a variation of the implementation of permutation feature importance (PFI)^23^ technique to evaluate the relative importance of each blood biochemistry marker to ensemble accuracy. The best performance by a DNN was MAE of 6.07 years in predicting chronological age and the ensemble learning produced MAE of 5.55 years. They identfied the 5 most important biomarkers for predicting human chronological age: albumin, glucose, alkaline phosphatase, urea and erythrocytes. Fischer *et al*.^24^ earlier identified four biomarkers: alpha-1-acid glycoprotein, albumin, very-low-density lipoprotein particle size, and citrate for predicting all-cause mortality by applying biomarker profiling via nuclear magnetic resonance spectroscopy. They also showed that these four biomarkers can predict healthy people that may be at a short-term risk of dying within 5 years from heart disease, cancer, and other illness. Findings from these studies suggest that particular biomarkers can be related to aging and mortality (for example albumin). Cole *et al*.^25^ studied the use of structural neuro-imaging MRI under a Gaussian process regression framework. The predicted age was identified as’brain-predicted age’ or brain age for short. They combined DNA-methylation with brain age and showed that the combination improved mortality risk prediction. On the contrary, they also combined brain age with grey matter and cerebrospinal fluid volumes, but that did not improve mortality risk prediction. Bobrov *et al*.^26^ proposed a DNN based model to estimate biological age using eye corners (called PhotoAgeClock). Their method resulted in an MAE of 2.3 years and 95% correlation with chronological age, however they did not consider biological age. Mamoshina *et al*.^27^ used a multilayer DNN model and showed population specific aging patterns for Canadian, Korean, and Eastern European subjects. Pyrkov *et al*.^7^ applied convolutional neural network (CNN) on a week long physical activity data (NHANES 2003–2006) measured per minute. They applied a four-layer one-dimensional CNN followed by two dense layers and a single unit layer to build the network. In a recent survey paper, Zhavoronkov *et al*.^28^ discussed recent advances and perspectives in using artificial intelligence for studying aging and longevity. Specifically, they discussed about work related to deep learning, transfer learning, and reinforcement learning. They also discussed different data modalities often used in biological age estimation such as biomedical images (e.g., MRI), genetic markers, and epigenetic attributes.

In this work, we consider the use of physical activity data for reliable estimation of human biological age. In particular, we consider the temporal nature of human locomotor activity as a key element in its use for analyzing biological age. Thus, rather than using CNN to estimate biological age as was done by Pyrkov *et al*.^7^, we apply a deep learning framework using Convolutional Long Short-Term Memory (ConvLSTM). Using the Cox proportional hazard model and Kaplan-Meier curves, we show comparative performance of our proposed biological age estimation methods with the existing deep learning approaches.

## Methodology

### Datasets

#### Activity dataset & preparation

We used physical activity data from the National Health and Human Nutrition Examination Surveys (NHANES) 2003–2004 and 2005–2006. NHANES uses a complex cluster design to sample members of the civilian USA population who are not institutionalized. Activity data is provided for a subset of NHANES participants. For these participants, NHANES provides physical (locomotor) activity for a 7-day continuous tracking of “activity counts” that is sampled every minute and recorded using a physical activity monitor (ActiGraph AM-7164 piezoelectric accelerometer (https://www.actigraphcorp.com/). For this work, we analyzed intensity of the physical activity, (also called device intensity value) recorded by the physical activity monitor. The devices were worn on the right hip by the individuals using an elastic belt. The combined dataset were of 14,631 study participants (7,176 in 2003–2004, and 7,455 in 2005–2006). Ethnicity included white, black, Mexican and others. Initially, we removed outlier samples with abnormally low (average activity count <50) or high (>5000) physical activity count. We excluded days with less than 200 minutes corresponding to activity states >0, similar to Pyrkov *et al*.^7^. Only participants with 4 or more days that passed this additional filter were retained, yielding a total of 7,104 individuals with 586 deaths (follow up in 2015). We have randomly chosen 6,000 individuals for training and 1,104 individuals for testing. All the algorithms are applied based on this partitioning of the dataset.

#### Anthropometric & biomarkers dataset

We also used NHANES 2003–2006 anthropometric and biomarker datasets. These were used to study the potential relationship between human physical activity and the biomarkers. Numerous prior studies^10,13^ established connections between biomarkers and biological aging. Also some recent anthropometry based body shape indices such as surface based body shape index (SBSI)^29^, and a new body shape index (ABSI)^30^ showed better prediction of all cause mortality because of their relation with aging. Anthropometric measurements included BMI, height, weight, and waist circumference. The biomarker dataset included information on systolic and diastolic blood pressure, albumin, high-density lipoprotein (HDL), low-density lipoprotein (LDL), and hemoglobin. After refining and merging the two datasets, we obtained 4,268 individuals common to both datasets, aged 18 to 84 years with 329 deaths (follow up in 2015).

### Data transformation & data representation

#### Data transformation

Given the nature of the time-series human locomotor data, with noise, and outliers, our first step is to apply some basic data transformation operations, such as smoothing and filtering. Given that the data is a sequential time series data of 7 days, we applied moving averages on the data while varying the window sizes. The values of the physical activity intensity ranges over a large magnitude and moreover the intensity values are always positive. Thus we applied log transformation on the data (since there are ‘0’ values in the original data, we added a negligible value (1) before applying log transformation). Another typical filtering operation on time series data is to apply moving averages that helps smoothing out the noise or outliers and also helps to find/extract long-term trends. We applied different types of moving averages (e.g., simple moving average (SMA), weighted moving average (WMA), and exponentially weighted moving average (EMA)). SMA is the unweighted mean of the previous *n* data points. Given that we have physical activity per minute *pa*_*i*_, for an *n*-length window, the simple moving average would be $p{a}_{sma}^{(i)}=\frac{1}{n}\mathop{\sum }\limits_{j=0}^{n-1}p{a}_{i+j}$. EMA is a first order infinite impulse response filter that applies weighting factors which decrease exponentially. The EMA for physical activity can be calculated recursively: $p{a}_{ema}^{(i)}=p{a}_{i}$, if i = 1 or $p{a}_{ema}^{(i)}=\alpha \ast p{a}_{i}+(1-\alpha )\ast p{a}_{i-1}$, if i ≠ 1 where the coefficient *α* denotes the weighting factor (ranges between 0 and 1). We have used $\alpha =\frac{2}{N+1}$. EMA has the best overall results. Moving averages were applied after the log transformation of the data. We also applied the Box-Cox transformation^31^. This a point transformation defined as *y*_*λ*_(*x*) = (*x*^*λ*^−1)/*λ*, if *λ* ≠ 0 or *y*_*λ*_(*x*) = log(*x*) if *λ* = 0.

#### Data representations

Pyrkov *et al*.^7^ applied the one dimensional data (10080 minutes) to a deep learning model to predict the biological age of a person. The basic idea was to use a one dimensional CNN architecture/model to extract features. This however, does not account for the temporal information in the data or the temporal pattern that a person might exhibit. For an improved representation of the potential temporal signals of biological age in the locomotor activity data, we consider the data as a temporal sequence of daily activity records. Each record is considered as a two dimensional matrix of size 24 × 60 capturing the locomotor activity for every hour and for each minute in the hour. The result is thus a three dimensional view of the locomotor activity data. This representation makes it easier to identify repeated temporal patterns in the data, which might provide cues to the functional or health status of the individual and thus to their biological age.

### LSTM estimation of biological age

**Figure 1:**
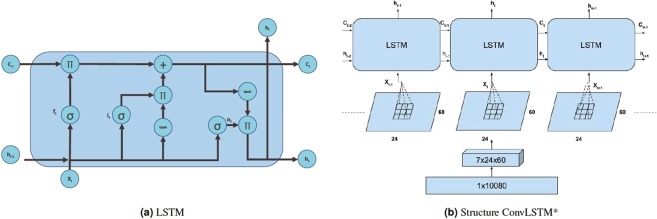
(**a**) Basic LSTM cell. Π denotes multiplication, + denotes addition, *σ* is sigmoid function, and *tanh* calculates hyperbolic tangent, (**b**) Transformation and inner structure of ConvLSTM*.

Given that we have a time series data with physical activity of a person in every minute for a week, it is natural to consider the use of a recurrent neural network to keep track of the time series data, in terms of memories of what the system has observed thus far. This is analogous to biological information/intelligence processing (i.e., a human processes information on incremental basis while keeping track of what he has processed so far). A recurrent neural network (RNN) has a ‘state’ that stores the information pertaining to what it has observed/processed thus far, and it processes sequential data through a number of iterations. So, an RNN is basically a neural network containing an internal loop and the state of the RNN is changed/reset between two sequences. The RNN, however, suffers from the problem of propagating vanishing gradients^32^. The Long Short-Term Memory (LSTM) is one of the most popular recurrent neural networks developed by Hochreiter and Schmidhuber^32^ that adds a way to carry information across sequences. This saves information for later, and prevents older signals from vanishing gradually. Figure 1(a) shows a basic LSTM cell. In the figure, *i*_*t*_ is the input gate, *f*_*t*_ is the forget gate, *c*_*t*−1_ is the previous cell output, *o*_*t*_ is the ouput gate, and *h*_*t*_ is the final state. LSTM updates for timestep *t* given input *x*_*t*_, and the previous state *h*_*t*−1_, and previous cell output *c*_*t*−1_. The LSTM updates are given as follows^32^:

$$\begin{array}{rcl}{i}_{t} & = & \sigma ({W}_{xi}{x}_{t}+{W}_{hi}{h}_{t-1}+{b}_{i})\\ {f}_{t} & = & \sigma ({W}_{xf}{x}_{t}+{W}_{f}{h}_{t-1}+{b}_{f})\\ {c}_{t} & = & {f}_{t}\ast {c}_{t-1}+{i}_{t}\ast tanh({W}_{c}{x}_{t}+{W}_{hc}{h}_{t-1}+{b}_{c})\\ {o}_{t} & = & \sigma ({W}_{xo}{x}_{t}+{W}_{ho}{h}_{t-1}+{b}_{o})\\ {h}_{t} & = & {o}_{t}\ast tanh({c}_{t})\end{array}$$

where *W*_*xi*_ is the weight matrix for input gate *i* and input *x*, and *b*_*i*_ is the bias for *i*. Whenever a new input comes, the first step is to decide what information is going to be discarded from the cell state. This is performed by the forget gate. The next step is to get what information will be in the cell state. The *c*_*t*_ first uses a sigmoid layer to decide which value to update and then a *tanh* layer provides a vector of new candiate values. Finally, the output will be based on the cell state with a filter (where a sigmoid decides the part of the output of cell state, and then finally the cell state is put through another *tanh* layer (output between −1 and 1) and multiplied by the output of the sigmoid gate).

Our basic approach to analyzing the human activity data is based on the idea of LSTM. We build on the concept of finding local patterns by applying the convolution operation of an image. Two major characteristics of convolutional neural networks (CNN) are that they can learn patterns in a hierarchical manner, and that the patterns learned are translation invariant (i.e., a learned pattern will be recognized anywhere). Our proposed method for estimating biological age is to apply the combination of LSTM with CNN to the human physical locomotor activity data. Note that this approach is different from the regular LSTM or CNN problem. Rather we take advantage of the structure in the sequence of 2D representations of the daily activities to learn valuable patterns from the activity data (which is very difficult using 1D CNN, LSTM, or DNN). We consider the data as a temporal 7(D) day information, where each day has 24 hours and an hour is 60 minutes. So to break it down, we represent it as a two dimensional information of 24 × 60 (HxM) minutes with a temporal information of 7 days. The three dimensional information is therefore, 7 × 24 × 60 (DxHxM) minutes of data. Figure 1(b) shows the representation described above. The image representation (HxM) of the 1D physical data for each day introduce different feature dimensions that cannot be learned easily using 1D CNN architecture. In particular, using the 24 × 60 matrix representation of physical activity, records at minute 1 and minute 61 are neighbors (when considered as an image or matrix form), while in a one dimensional sequential view they are 60 timesteps apart. Two important factors here are that the spatial structure is changed, and that the sequence of two-dimensional information is very different from that of the original one-dimensional time series (especially, the information gathered from the 1D CNN and 2D CNN).

**Figure 2:**
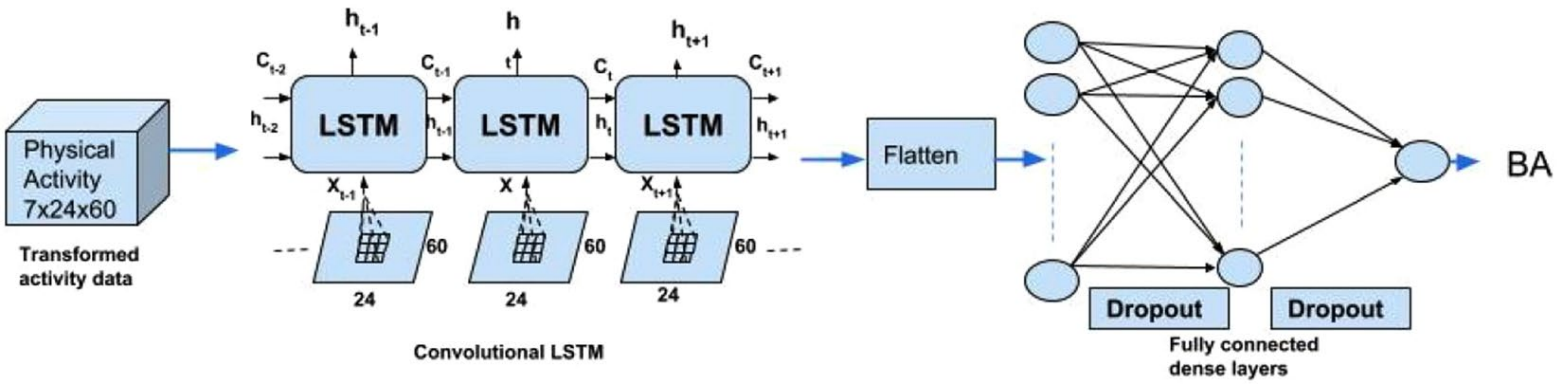
Architecture of the proposed ConvLSTM* deep learning method for biological age estimation using human locomotor activity data.

Our approach to combining LSTM and CNN builds on the idea of ConvLSTM proposed by Shi *et al*.^33^. Under ConvLSTM, the convolution structures are applied both at the input-to-state transition and at the state-to-state transitions. The ConvLSTM differs from simple CNN + LSTM in that, for CNN + LSTM, the convolution structure (CNN) is applied as the first layer and sequentially LSTM layer is applied in the second layer. Our approach also differs from ConvLSTM in that, we do not attempt to predict the next 2D matrix of physical activity for an individual, rather our goal is to estimate the biological age. Thus we concatenate two more fully connected dense layers and finally a single unit of neuron without activation to build up a scalar regression that estimates the biological age. We call our approach ConvLSTM*. Figure 2 shows the architecture of the proposed ConvLSTM* model. We also apply 1D CNN^7^ and 1D DNN (see Supplementary Materials) on the 1D time series dataset to predict biological age. We compare the results from these three deep neural network models.

### Statistical analysis

We analyzed the data separately for male and female subjects, and for their combination. Table 1 shows the key biomarker attributes used in this study. Table 1 also includes some important anthropometric measurements such as body mass index (BMI), surface-based body shape index (SBSI), waist-to-height ratio (WHtR), and average physical activity. SBSI was computed following Rahman and Adjeroh^29^. We have used the following metrics to evaluate accuracy: (1) Pearson correlation coefficient between *x* and *y*: $\rho (x,y)=\frac{{\sum }_{i=1}^{N}\,({x}_{i}-\bar{x})({y}_{i}-\bar{y})}{\sqrt{{\sum }_{i=1}^{N}\,{({x}_{i}-\bar{x})}^{2}}\sqrt{{\sum }_{i=1}^{N}\,{({y}_{i}-\bar{y})}^{2}}}$, where *x* and *y* are different attributes, *N* is number of samples. (2) Mean absolute error $MAE=\frac{1}{N}\mathop{\sum }\limits_{i=1}^{N}\,|{y}_{i}-{\hat{y}}_{i}|$, where *y*_*i*_ is the original value and ${\hat{y}}_{i}$ is the estimated value. In this work MAE shows the average change/error between the chronological age and the estimated age. (3) R-squared distance also known as coefficient of determination, ${R}^{2}=1-\frac{{\sum }_{i=1}^{N}\,{({y}_{i}-{\hat{y}}_{i})}^{2}}{{\sum }_{i=1}^{N}\,{({y}_{i}-\bar{y})}^{2}}$, where *y*_*i*_ is the original value, ${\hat{y}}_{i}$ is the estimated value, and $\bar{y}$ is the mean of *y*, and (4) Root mean square error (RMSE), $RMSE=\sqrt{\frac{1}{n}{{\rm{\Sigma }}}_{i=1}^{n}{({y}_{i}-{\hat{y}}_{i})}^{2}}$, where *y*_*i*_ is the original value and ${\hat{y}}_{i}$ is the estimated value.

**Table 1:** Key attributes for study participants using the NHANES (2003–2006) dataset.

	All (N = 4268)	Female (N = 2094)	Male (N = 2174)
**Attributes**	Average ± SD	Average ± SD	Average ± SD
C-reactive protein (mg/dL)	0.37 ± 0.82	0.41 ± 0.7	0.33 ± 0.92
Glycated hemoglobin (%)	5.45 ± 0.89	5.37 ± 0.76	5.53 ± 0.99
Serum Albumin (ug/mL)	4.23 ± 0.40	4.10 ± 0.42	4.36 ± 0.33
Total Cholesterol (mg/dL)	197.96 ± 42.56	202.65 ± 43.76	193.44 ± 40.88
Serum Urea Nitrogen (mg/dL)	12.34 ± 5.49	11.2 ± 5.22	13.43 ± 5.52
Serum Alkaline Phosphatase (U/L)	70.69 ± 27.45	69.09 ± 28.48	72.24 ± 26.33
Systolic blood pressure (mm Hg)	123.68 ± 20.49	121.58 ± 22.84	125.71 ± 17.7
Diastolic blood pressure (mm Hg)	68.33 ± 13.76	66.74 ± 14.13	69.86 ± 13.22
Pulse (60 sec)	71.95 ± 12.55	74.89 ± 12.49	69.11 ± 11.95
High density lipoprotein (mg/dL)	55.95 ± 16.59	62.42 ± 16.99	49.72 ± 13.54
Hemoglobin (g/dL)	14.39 ± 1.5	13.51 ± 1.22	15.25 ± 1.23
Lymphocyte percent (%)	29.8 ± 9.00	29.54 ± 8.99	30.06 ± 9.00
White blood cell count (SI)	7.31 ± 2.87	7.47 ± 2.72	7.15 ± 3.00
Hematocrit (%)	42.58 ± 4.44	39.9 ± 3.56	45.15 ± 3.61
Red blood cell count (SI)	4.71 ± 0.52	4.43 ± 0.41	4.98 ± 0.47
Platelet count (% SI)	266.38 ± 68.3	280.54 ± 70.7	252.75 ± 62.97
Body mass index (BMI) (*kg*/*m*^2^)	26.44 ± 4.61	26.09 ± 4.89	26.77 ± 4.3
Surface based body shape index (SBSI)	0.12 ± 0.01	0.12 ± 0.01	0.12 ± 0.01
Waist-to-height ratio (WHtR)	0.56 ± 0.08	0.56 ± 0.08	0.55 ± 0.08
Age (years)	46.3 ± 20.18	44.91 ± 20.25	47.65 ± 20.02
Average physical activity	156.78 ± 119.08	136.88 ± 111.66	175.94 ± 122.84

To evaluate the estimated biological ages, we have applied two statistical models for validation and comparisons of BA algorithms, namely, Cox proportional hazard model (Cox PH) and Kaplan-Meier (KM) curves. We calculated the hazard ratio and corresponding p-value for Cox PH model using function “coxph” from R package “survival”. Log-rank test was performed to quantify the KM plots. To perform log-rank test, we used “survdiff” function and calculated the chi-square distance and the corresponding p-value. In all cases, for p-value smaller than 0.05 the result is considered significant. In this work, for the Cox PH and KM curves, we have used follow-up time for the time parameter in the models. Table 2 shows the correlation coefficient between average physical activity and the biomarkers. These results are based on the individuals that have both biomarkers and physical activity data available. The sample mean of age was 46.30 years, mean body mass index (BMI) is 26.44 *kg*/*m*^2^, and mean surface based body shape index (SBSI) of 0.12 (see Table 1).

**Table 2:** Correlation between average physical activity, chronological age, and blood biomarkers.

	Average Physical Activity		Chronological Age	
	Correlation	p-value	Correlation	p-value
C-reactive protein	−0.083	5.29E-08	0.048	1.67E-03
Glycated hemoglobin	−0.086	1.92E-08	0.340	3.15E-116
Serum Albumin	0.167	3.65E-28	−0.090	4.46E-09
Total Cholesterol	−0.034	2.74E-02	0.182	5.94E-33
Serum Urea Nitrogen	−0.059	1.20E-04	0.457	3.09E-21
Serum Alkaline Phosphatase	−0.068	9.28E-06	0.062	5.03E-05
Systolic blood pressure	−0.110	4.75E-13	0.535	1.32E-31
Diastolic blood pressure	0.049	1.34E-03	0.099	1.12E-10
Pulse	−0.084	3.80E-08	−0.211	2.88E-44
High density lipoprotein	−0.024	1.19E-01	−0.014	3.64E-01
Hemoglobin	0.150	9.04E-23	−0.024	1.13E-01
Lymphocyte percent	0.059	1.07E-04	−0.066	1.68E-05
White blood cell count	−0.067	1.06E-05	−0.053	5.33E-04
Hematocrit	0.147	3.94E-22	−0.011	4.86E-01
Red blood cell count	0.151	4.74E-23	−0.133	2.41E-18
Platelet count	−0.038	1.25E-02	−0.116	3.31E-14
Chronological age	−0.193	2.0E-16		

All statistical analyses were performed using the R Language, Ver. 3.3.5 (The R Foundation for Statistical Computing, Vienna, Austria). The following packages were used: survival, gtools, ggplot2, tidyverse, Tensorflow, keras, reticulate, e1071, randomForest, matrixStats, technical trading rules (TTR).

## Results

### Human locomotor activity is associated with chronological age

**Figure 3:**
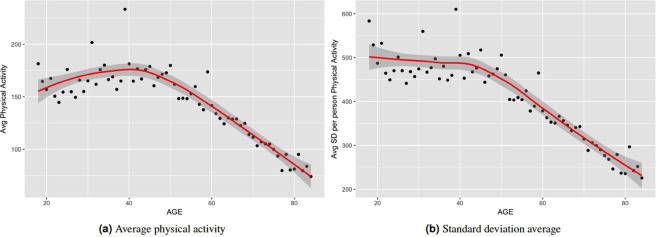
Variation of average physical activity with age. Values plotted for individuals grouped by year of age.

To motivate our study of deep learning methods for biological age estimation using the locomotor physical activity data, we first investigated whether there is any discernible association between physical activity and chronological age (which is easier to assess than biological age). On average, physical activity has a correlation coefficient of −0.19 (p-value = 0.00) with chronological age (see Tables 2 and S4). To further investigate the relation between physical activity and chronological age, we have grouped subjects in the physical activity dataset based on their age ranging from 18 to 84. For subjects at a given age, we compute their average physical activity. Figure 3(a) shows the results. We also calculate the standard deviation (SD) of physical activity for each person. Similarly, we calculate average of SD of physical activity at each given age group. Figure 3(b) shows the average SD for each age in our dataset. Average physical activity goes up from age 18 to 45. After that we observe a generally linear decline of average physical activity from age 46 to 85 years. However, standard deviations do not vary significantly from age 18 to 45 although they show a slightly downward trend. Then it goes down linearly till age 85. We observe a pattern of reduced physical activity on average with increasing age after 45. Standard deviation of physical activity on average also reduces after 45 years. There seems to be a region around age 45 years, after which both average physical activity and SD of physical activity reduce linearly with age.

### ConvLSTM* estimated BA acceleration using physical activity data is associated with BA from biomarkers

Biomarkers have been used frequently to estimate biological age in prior works^10,13^. We refined and merged the physical activity dataset with biomarker dataset, and obtained 4268 common individuals. This allowed us to compare results and observations using the proposed ConvLSTM* using activity data with those from traditional biomarkers.

#### Association between locomotor activity with biomarkers

**Figure 4:**
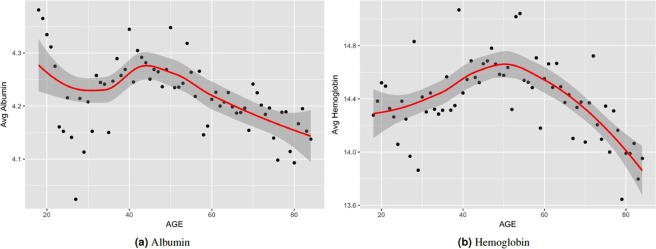
Variation of two biomarkers (albumin, and hemoglobin) with age. Values plotted are the average measurements for individuals grouped by their age.

To further investigate the potential effectiveness of using the locomotor activity data for predicting biological age, we analyzed the correlation of known biomarkers of aging with the average physical activity. For biomarkers of aging, we considered 16 of the biomarkers available in NHANES, namely, C-reactive protein, glycated hemoglobin, albumin, total cholesterol, urea nitrogen, alkaline phosphatase, systolic blood pressure, diastolic blood pressure, pulse, high density lipoprotein, hemoglobin, lymphocyte percent, white blood cell count, hematocrit, red blood cell count, platelet count. Subsets of these have been used in earlier work as key biomarkers of biological age^9,10,13,17^. Table 2 shows the correlation between the average physical activity and the biomarkers used (see also Table S4). The table shows the correlation using direct measurements for Pearson’s *ρ* and corresponding p-value of the correlation. Albumin has the highest correlation (*ρ* = 0.17, p-value = 0.00). Red blood cell count, and hemoglobin each has correlation of 0.15 (p-value = 0.00). Interestingly, high-density lipoprotein (HDL) has a very low correlation with physical activity (*ρ* = −0.024, p-value = 0.119). This result is similar to the study by Levine *et al*.^9^ where they showed that HDL has a little correlation (*ρ* = 0.026) with chronological age. Figure 4 shows how the two most correlated biomarkers (Albumin (*ρ* = 0.17), and Hemoglobin (*ρ* = 0.15)) vary with chronological age on average. From Figs 3 and 4 we can observe that in general the average physical activity has a similar trend with the biomarkers (Albumin and Hemoglobin) with respect to age. In Fig. 4(a) average albumin goes down from ages 18 to 35 then goes up till about 45; from ages 45 to 84 albumin consistently goes down on average. Hemoglobin, however, goes up on average, from age 18 to 50, and then consistenly goes down till age 84. This is similar to the observation of Belsky *et al*.^10^ on the pace of aging. They showed that the pace of aging can be rapid for individuals who are biologically older than their peers, i.e. a 38 year old with a biological age of 40 will age faster than a 38 year old with biological age of 38 years. Figure 3 also shows a similar property for average physical activity where the decline in physical activity starts at around age 45. Figure S3 shows the correlation between physical activity and the blood biomarkers for each age. This shows the trend in correlation for each age, and thus avoids potential problem of averaging positive and negative correlations of different age ranges. For instance, diastolic blood pressure has positive correlation with activity for age ranges 18–40 and 60–84, but negative correlation in between (ages 41–59). On the other hand, urea nitrogen starts out with high positive correlation which decreases progressively with increasing age. By age 70 and above it exhibits negative correlation with physical activity.

#### Similarity between estimated biological age from biomarkers and ConvLSTM* estimated biological age from locomotor activity

**Figure 5:**
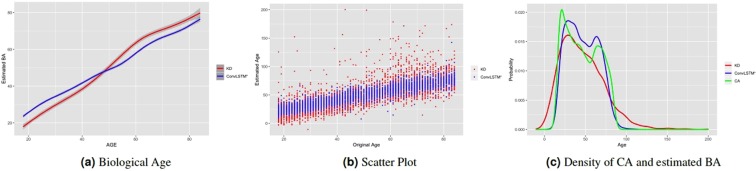
Similarity between average biological estimated age using biomarkers using KD method and physical activity using ConvLSTM* over the age range 18–84. (**a**) Estimated biological age (smoothed using generalized additive model) against chronological age, (**b**) Estimated biological ages against chronological age, (**c**) distribution of original chronological age (CA) and estimated biological age using biomarkers (KD method) and physical activity (ConvLSTM* method). These results are for common individuals in both the biomarker and physical activity datasets.

Figure 5(a) shows the estimated biological age using the Klemera and Doubal (KD)^12^ method applied on the biomarker data and the corresponding estimated biological age using the proposed ConvLSTM* deep learning method applied on the locomotor activity data, for the 4268 individuals that have both physical activity and biomarker data available. Within the age range from 18 to 84 we took the individuals for each given age and calculated the mean of the estimated age. Both KD and ConvLSTM have similar results, with each producing BA estimates that increase consistently (generally linearly) with chronological age. At around age 47, we observe a crossover of the estimated BAs. This is pertinent with both the linear decrease of average physical activity in Fig. 3 and linear decrease of albumin and hemoglobin in Fig. 4 after age 45. Thus, it might be possible to estimate biological age based on physical activity of an adult individual. Figure 5(b) shows the scatter plot for the results in Fig. 5(a). We observe that the KD estimated ages are more scattered and vary more widely while ConvLSTM* estimated ages are more confined within a smaller range. This is also justified by the mean absolute error values of the two methods. However, lower MAE may not always lead to better BA estimates. In fact, the large range of BA estimates generated by KD may have positive impacts in its use in survival/mortality modeling. Moreover, as we are minimizing the mean square error (MSE) as our loss function, our approach may suffer from the problem of “regressing to the mean^19^”. Figure 5(c) shows the respective age distributions using the estimated BA from KD method on biomarker data, the proposed deep learning ConvLSTM* method on the locomotor activity data, and the chronological age. We observe that ConvLSTM* and the chronological age are closely aligned while KD estimated values are very widely spread (estimated age occasionally fit to very high and very low values, at times up to 150 years).

Following Mitnitski *et al*.^11^ biological age acceleration is defined simply as Δ = *CA*−*BA*, where CA denotes chronological age and BA denotes biological age. However, here we introduce the normalized form which we call normalized biological age acceleration (NBAA), denoted $\eta =\frac{{\rm{\Delta }}}{CA}=\frac{CA-BA}{CA}$. This normalization is performed to reduce the effect of of large Δs. For example, a Δ value of 5 could have different biological or health implications for an 18 year old (≈28% difference) and for a 70 year old (≈7% difference). Our observation is that, overall, using *η* values provided more improvements in the results when compared to using Δ values.

### Deep learning biological age using ConvLSTM* on physical activity data leads to improved modeling of all-cause mortality

Biological age is a quantitative measure which is expected to provide some general indication of the health/functional status of an individual. Numerous approaches have used the idea of the association of physiological variables (biomarkers, activity) for estimating BA^7,10,25,34^. However, given a new data modality, such as the human locomotor activity data studied in this work, a different model may be needed. Thus, we built a learning model using deep convolutional long short term memory (ConvLSTM) that is trained to estimate the BA. We applied two-dimensional ConvLSTM on the data, taking advantage of both convolution and LSTM at the same time for extracting features rather than using the one-dimensional time series data. The proposed network architecture is shown in Fig. 2. To evaluate how well the estimated BA using the proposed deep learning ConvLSTM* approach on locomotor activity data captures the functional status of the subjects, we considered how the estimated BA relates to health risks. In particular, we studied the association of all-cause mortality with the normalized biological age acceleration (*η*) using the estimated BA models. Unless otherwise indicated, reported results are based on the larger activity dataset with 7,104 individuals.

#### Cox PH model

We used Cox proportional hazard modeling (Cox PH)^35,36^ and Kaplan-Meier (KM) curves^37^ to quantify the association of the proposed ConvLSTM* estimated BA with all-cause mortality. Under the Cox model, the relationship between hazard and the covariates is described by considering the logarithm of the hazard as a linear function of the variables. Here we calculate the hazard ratio (HR) for each BA estimation algorithm. First we estimate biological age (BA) using 1D CNN^7^, DNN, CNN + LSTM, and ConvLSTM* models, and then we calculate $\eta =\frac{CA-BA}{CA}$ for each BA prediction algorithm. The DNN and CNN + LSTM models were developed and implemented in this work. The ConvLSTM* model is parameterized using the data transformation parameter, *λ*. Table 3 shows the results for all the approaches.

**Table 3:** Results of the Cox proportional hazard (Cox PH) model applied on the normalized biological age acceleration *η* = (*CA*−*BA*)/*CA* for estimated biological ages.

	HR	p-value
1D CNN^7^	1.05 (1.04, 1.07)	1.63E-11
DNN	1.07 (1.06, 1.09)	1.75E-19
CNN + LSTM (*λ* = 0.9)	1.05 (1.05, 1.08)	1.65E-11
ConvLSTM* (*λ* = 1)	1.06 (1.04, 1.07)	1.38E-14
ConvLSTM* (*λ* = 0)	1.07 (1.05, 1.08)	7.81E-17
ConvLSTM* (*λ* = 0.9)	1.05 (1.04, 1.07)	1.74E-11

We applied *η* as the co-variate in the Cox model. We observe that for 1D CNN, and DNN, the HR value is 1.05, and 1.07 respectively. And the proposed ConvLSTM based method has similar results (ConvLSTM* (*λ* = 1), HR = 1.06; ConvLSTM* (*λ* = 0), HR = 1.07; ConvLSTM* (*λ* = 0.9), HR = 1.05). From the perspective of Cox PH model, we found that the proposed ConvLSTM* based BA prediction method has similar performance compared to the other methods. Best overall results were obtained using ConvLSTM* (*λ* = 0), with HR = 1.07 (p-value 7.81E-17), and DNN with HR = 1.07 (p-value 1.75E-19) using the normalized biological age acceleration, *η*. For the common subset of data which contains both biomarker attributes and physical activity of individuals we applied CoxPH model on the estimated age by KD method using biomarkers, and deep learning methods using physical activity methods. Using KD estimated biological age we obtained HR = 1.30 (p-value 5.08E-1). For comparison, on this common data set, ConvLSTM* (*λ* = 0) produced HR = 1.09 (p-value 3.02E-14).

#### KM plots and LogRank

**Figure 6:**
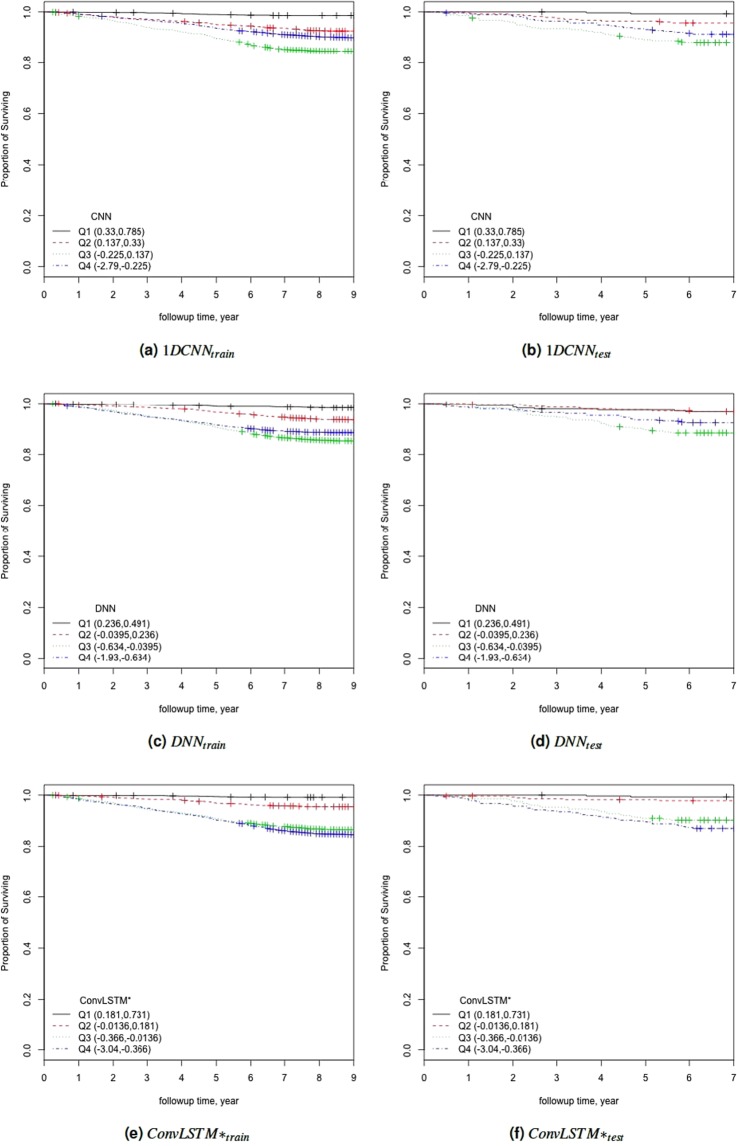
The Kaplan Meier curves for estimated biological ages (BA) based on the physical activity applying $\eta =\frac{CA-BA}{CA}$ for both training (**a,c,e**), and test (**b,d,f**) data. Q1, Q2, Q3, and Q4 denote 1st, 2nd, 3rd, and 4th quartiles, respectively. The number of individuals in each Q is 276 (test data). Left column corresponds to results for training, while right column corresponds to results for testing.

To further study the performance of the estimated BAs, we analysed the Kaplan-Meier (KM) survival curves obtained using the quantile factored NBAA $(\eta =\frac{CA-BA}{CA})$. Figure 6 shows the KM plots for the BA estimation algorithms (Fig. S1 shows the same plots with confidence interval). A given variable is a good mortality predictor if the Kaplan-Meier curves are easily distinguishable (more distance between them), and the variable captures the survival rates from low to high levels, with less crossing between curves. In general, each method of predicting biological age perform relatively well in distinguishing the proportion of survivors. Among the deep learning BA estimation methods, the Pyrkov *et al*. approach^7^ using 1D CNN performed better than using the direct DNN model on the 1D data. However, once again, the proposed ConvLSTM* approach (using *λ* = 1) produced an improved result when compared with the Pyrkov *et al*.'s method^7^. To further quantify the performance, we used the log-rank test to compare the survival distributions obtained using the different BA algorithms. The log-rank test compares the Kaplan-Meier curves to check if they are statistically equivalent. The output of the test is a *χ*^2^-distance, and the p-value associated with the distance. Higher *χ*^2^-distances and low p-values indicate a better separation between the curves, and hence a better performance in mortality modeling. The difference among the biological age estimation methods is more evident using quantitative measures, e.g., the *χ*^2^-distance between their respective KM curves, as captured by the log-rank test (Table 4). ConvLSTM* (*λ* = 1) estimated biological age has the highest *χ*^2^-distance followed by CNN + LSTM and ConvLSTM* (*λ* = 0, 0.9). For the common subset of data which contains both biomarker attributes and physical activity of individuals we applied log-rank test on the estimated age by KD method using biomarkers. We obtained a *χ*^2^-distance of 88.49 (p-value 4.62E-8). For comparison, on this common data set, ConvLSTM* (*λ* = 0) produced *χ*^2^-distance = 49.15 (p-value 1.21E-10).

**Table 4:** Results of the log-rank test applied on the normalized biological age acceleration *η* = (*CA*−*BA*)/*CA* using the estimated biological ages.

	Chi-Sq	p-value
1D CNN^7^	33.60	2.41E-07
DNN	22.10	6.22E-05
CNN + LSTM (*λ* = 0.9)	48.19	1.94E-10
ConvLSTM* (*λ* = 1)	48.28	1.86E-10
ConvLSTM* (*λ* = 0)	44.38	1.25E-09
ConvLSTM* (*λ* = 0.9)	24.15	2.33E-05

## Discussion

In this work we have investigated deep learning approaches on the NHANES (2003–2006) locomotor physical activity data. We estimate biological age (BA) based on the physical activity and chronological age (CA). To quantify how well the estimated biological age captures the health risk, we apply the Cox proportional hazard model with all-cause mortality. The deep learning models such as DNN, CNN, and ConvLSTM were trained to exploit the dependence of the physiological/activity changes with age. In all cases, the deep learning approaches were trained to minimize the mean squared error (MSE) between estimated BA and CA, in every epoch.

### Parameter choices

We tested the performance of smoothening/filtering the original 1D activity data using different moving averages (simple moving average (SMA), weighted moving average (WMA), and exponential moving average (EMA)). We observed that EMA provided the overall best result. To test the impact of window size (N), we performed experiments using different values. We have considered N = 1, 2, 4, 8, 10, 16, 20, 25, 30, 35, 40. Table S1 shows the impact of the window size used for calculating the moving averages. We show the performance using different window sizes (N) based on two machine learning algorithms, namely, support vector machine (SVM) and random forest (RF). Table S1 shows the variation of window size of exponential moving averages using the Box-Cox transformation. The performance criteria for choosing the window size was to get lower MAE, higher R-squared distance, and higher correlation. From these results, we selected N = 35 as the best overall window size (*R*^2^ = 0.48 for SVM). Table S2 shows the impact of *λ* for Box-Cox transformation. Best results were obtained with *λ* = 0.9 (*R*^2^ = 0.56 for SVM using N = 35). We have reported results for ConvLSTM* using three values of *λ*; *λ* = 1 (raw, with no transformation), *λ* = 0 (log transformation), and *λ* = 0.9.

For ConvLSTM* layer we have used 128 filters, a kernel size of 3 ×3 with a “ReLU” activation function. The first dense layer has 256 filters and second has 128 filters. Weight initialization was performed by Glorot and Bengio normal initialization^38^, 30% dropout was performed after each dense layer. We have tried different optimizers such as rmsprop^39^, Adam^40^, and Nadam^41^. Based on the empirical results, we have selected Adam optimizer for this work. Circular padding was used for CNN. Mean square error (MSE) was used for loss function. We used Keras (https://keras.io/) library with Tensorflow (https://www.tensorflow.org/) in the backend to build the deep learning models. All experiments were performed using a NVIDIA 1080Ti graphics processing unit (GPU) running on a Ubuntu 16.04 (operating system) machine with Intel core-i7 processor and 32GB RAM.

### Impact of gender

Results reported so far are from a single model that does not consider gender differences. That is, the same model is used for both female and male. However, gender is expected to have an influence on the performance of an age estimation scheme^42–44^. Table 5 shows the results for separate gender specific models. We observe that, for gender specific models, applying normalized biological age acceleration *η* = (*CA*−*BA*)/*CA* using estimated BA have higher hazard ratios than using chronological age. Moreover, for *λ* = 0 and *λ* = 1, using chronological age, the p-values are not significant. Using a separate model for male resulted in higher HR values (for each *λ*). However, using a separate model for female did not improve the hazard ratios when compared with using the single model for all.

**Table 5:** Results of the Cox Proportional Hazard model (CoxPH) applied on the normalized biological age acceleration *η* = (*CA*−*BA*)/*CA* using separate models for female and male subjects.

	Female		Male	
	HR	p-value	HR	p-value
ConvLSTM* (*λ* = 1)	1.06 (1.04, 1.08)	5.16E-08	1.05 (1.03, 1.07)	1.45E-05
ConvLSTM* (*λ* = 0)	1.05 (1.03, 1.08)	2.84E-06	1.05 (1.03, 1.07)	2.36E-08
ConvLSTM* (*λ* = 0.9)	1.06 (1.04, 1.09)	1.87E-06	1.04 (1.02, 1.06)	1.13E-05

**Figure 7:**
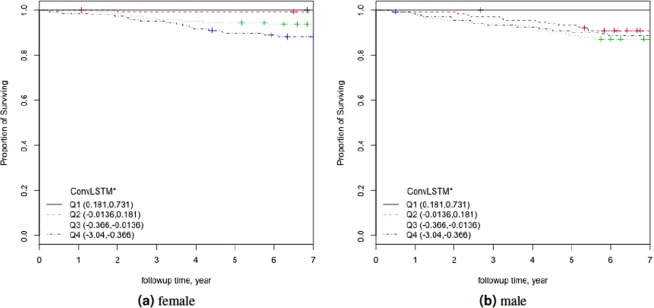
The Kaplan Meier curves for applying $\eta =\frac{CA-BA}{CA}$ on the physical activity (**a**) female, and (**b**) male. Q1, Q2, Q3, and Q4 denote 1st, 2nd, 3rd, and 4th quartiles, respectively.

Figure 7 shows the KM plots for gender specific models applying *η* = (*CA*−*BA*)/*CA* (ConvLSTM estimated BA) factored into quartiles. Using separate male model’s KM plots were of similar nature in comparison with the combined KM plots. See Fig. 6(e). However, for separate female model, the KM curve is slightly different although all the quartiles are well separated. To further quantify these results, we perform log-rank test on the models. Table 6 shows the results for log-rank test for separate female and male models. Similar to the results of Cox PH models and KM curves, log-rank test also show better results for male model with higher *χ*^2^ values for all the *λ* variations.

**Table 6:** Results of Log-rank tests applied on the normalized biological age acceleration *η* = (*CA*−*BA*)/*CA* using separate models for female and male subjects.

	Female		Male	
	Chi-Sq	p-value	Chi-Sq	p-value
ConvLSTM* (*λ* = 1)	29.33	1.91E-06	16.97	7.18E-04
ConvLSTM* (*λ* = 0)	25.69	1.11E-05	18.23	3.94E-04
ConvLSTM* (*λ* = 0.9)	17.83	4.78E-04	19.15	2.54E-04

### Variations on biological age acceleration

In this work, so far we have used *η* = (*CA*−*BA*)/*CA*. In previous work^11^ age acceleration was defined as Δ = *CA*−*BA*. We introduce the normalized form to reduce the effect of low values or high values of CA. However, because of the fitting minimization of mean square error (MSE) as the loss function, this definition of *η* may still suffer from the “regressing to the mean” problem^19^. To solve the problem we introduce variations of biological aging acceleration. We calculate the difference between individuals’ biological age and the corresponding age, and gender matched cohort average. Thus we define Δ_*g*_ = *BA*_*g*_−*BA*, and ${\eta }_{g}=\frac{B{A}_{g}-BA}{CA}$. Figure S2 shows the distribution of age acceleration for *η*, and *η*_*g*_ over age groups. We notice that *η*_*g*_ have better distribution for all age groups. For all the age groups except age group 1 (≤30) we observe a shift to the left from *η* to *η*_*g*_. Table S4 shows the correlation of the average physical activity (PA Avg), chronological age, variations of aging acceleration (*η*, *η*_*g*_) with respect to the biomarkers used in this work. *η*_*g*_ have higher correlation with most biomarkers. Thus it may be the case that *η*_*g*_, the biological aging acceleration calculated based on the age and gender matched cohort average is more powerful in exposing the relationship between biomarkers and aging.

### Connection with general health status

Another way to investigate the performance of the proposed ConvLSTM* in capturing health risks is to consider their possible relationship with known indicators of health risk or how the estimated biological age differentiates between subjects with known diseases and those without. Below we consider these two perspectives in evaluating a BA estimation method.

#### Relationship with known health indices

For general indices of health status, we can consider the body mass index (BMI), waist to height ratio (WHtR), or the more recently introduced surface based body shape index (SBSI)^29^ or ABSI^30^. In particular, we studied the variation of the proposed normalized biological age acceleration (NBAA, denoted *η*) computed using the estimated BA from ConvLSTM* with variations in the WHtR, and in SBSI categories. Earlier studies by Morkedal *et al*.^45^ have shown that the WHtR is a better measure of health status when compared with BMI. Rahman and Adjeroh^29^ made a similar observation on the superiority of SBSI over BMI. We have also observed the performance of ConvLSTM* with respect to the surface based body shape index (SBSI)^29^ quartiles. Table 7 shows the log-rank test on the SBSI quartiles. The results are shown using *η*, for each SBSI category. We observe that, in general the *χ*^2^ values increase from first quartile to fourth quartile. However, the increase is not monotonic for all the variations of *λ*. For example, the *χ*^2^-distance decreased from *Q*_2_ (7.75) to *Q*_3_ (2.89) and then increased for *Q*_4_ (15.83) for *λ* = 1, other variations (*λ* = 0, 0.9) follow a similar trend. We observe a similar trend for male-only models as well. Using female-only models, the *χ*^2^-distances increased monotonically for *λ* = 1, 0. The performance of ConvLSTM* with respect to the waist-to-height ratio (WHtR) quartiles is of similar nature to the results on SBSI quartiles (see Table 8). We observe that, *χ*^2^-distances increase from *Q*_1_ to *Q*_4_ for *λ* = 0, 0.9. However, the *χ*^2^-distances for the fourth quartile are not always greater than those for the third quartile, although they are greater than both first and second quartile. We also observed the relationship between the variants of biological aging acceleration with SBSI. Performance of *η*_*g*_ is generally similar with the performance of *η* in Table 7 for each *λ* (*λ* = 0, 0.9, 1) using all, female-only, and male-only models. For *λ* = 0, *χ*^2^-distance increased monotonically from *Q*_1_ (8.74) to *Q*_4_ (65.76), and for *λ* = 0.9, the *χ*^2^-distance increased from *Q*_1_ (6.70) to *Q*_4_ (26.58) for female-only model. In general, we observed significant differences in the *χ*^2^-distances between Q1 and Q4, and also between (Q1/Q2) and (Q3/Q4). This was the case for both SBSI and WHtR.

**Table 7:** Log rank results applying $(\eta =\frac{CA-BA}{CA})$ for different SBSI categories. Results are shown for model with all subjects, female-only, and male-only separately. Q1, Q2, etc. denote 1st quartile, 2nd quartile, etc.

	ConvLSTM*	*SBSI*_*Q*1_		*SBSI*_*Q*2_		*SBSI*_*Q*3_		*SBSI*_*Q*4_	
		Chi-sq	p-value	Chi-sq	p-value	Chi-sq	p-value	Chi-sq	p-value
ALL	ConvLSTM* (*λ* = 1)	3.03	3.87E-01	7.75	5.14E-02	2.89	4.09E-01	15.83	1.23E-03
	ConvLSTM* (*λ* = 0)	18.66	3.21E-04	5.78	1.23E-01	29.01	2.23E-06	61.52	2.78E-13
	ConvLSTM* (*λ* = 0.9)	13.25	4.12E-03	8.57	3.55E-02	13.37	3.90E-03	38.01	2.81E-08
Female	ConvLSTM* (*λ* = 1)	3.58	3.11E-01	4.86	1.82E-01	10.28	1.64E-02	12.88	4.90E-03
	ConvLSTM* (*λ* = 0)	10.34	1.59E-02	11.35	9.95E-03	24.25	2.21E-05	65.76	3.45E-14
	ConvLSTM* (*λ* = 0.9)	7.69	5.28E-02	6.25	1.00E-01	11.52	9.23E-03	26.58	7.23E-06
Male	ConvLSTM* (*λ* = 1)	2.55	4.67E-01	7.69	5.29E-02	4.38	2.23E-01	3.71	2.94E-01
	ConvLSTM* (*λ* = 0)	5.07	1.67E-01	1.58	6.63E-01	3.02	3.89E-01	8.00	4.60E-02
	ConvLSTM* (*λ* = 0.9)	13.86	3.10E-03	3.56	3.14E-01	12.4	6.14E-03	27.84	3.92E-06

**Table 8:** Log rank results applying normalized biological age acceleration $(\eta =\frac{CA-BA}{CA})$ for different WHtR quartiles. Results are shown for model with all subjects, and for separate models for females and males. Q1, Q2, etc. denote 1st quartile, 2nd quartile, etc.

	ConvLSTM*	*WHtR*_*Q*1_		*WHtR*_*Q*2_		*WHtR*_*Q*3_		*WHtR*_*Q*4_	
		Chi-sq	p-value	Chi-sq	p-value	Chi-sq	p-value	Chi-sq	p-value
ALL	ConvLSTM* (*λ* = 1)	9.74	2.09E-02	1.96	5.81E-01	23.14	3.78E-05	7.74	5.17E-02
	ConvLSTM* (*λ* = 0)	32.8	3.56E-07	26.18	8.75E-06	33.98	2.00E-07	35.04	1.20E-07
	ConvLSTM* (*λ* = 0.9)	15.01	1.81E-03	23.94	2.57E-05	24.93	1.60E-05	26.73	6.70E-06
Female	ConvLSTM* (*λ* = 1)	6.7	8.20E-02	4.86	1.82E-01	11.7	8.48E-03	3.39	3.36E-01
	ConvLSTM* (*λ* = 0)	20.63	1.26E-04	31.25	7.52E-07	24.65	1.83E-05	25.58	1.17E-05
	ConvLSTM* (*λ* = 0.9)	9.38	2.47E-02	15.34	1.55E-03	11.83	8.00E-03	12.65	5.47E-03
Male	ConvLSTM* (*λ* = 1)	11.35	9.96E-03	5.45	1.42E-01	19.09	2.62E-04	9.73	2.10E-02
	ConvLSTM* (*λ* = 0)	13.02	4.60E-03	11.26	1.04E-02	7.29	6.31E-02	7.32	6.24E-02
	ConvLSTM* (*λ* = 0.9)	19.67	1.99E-04	18.96	2.79E-04	20.01	1.69E-04	22.09	6.26E-05

#### Relation with disease status

We also considered whether the proposed measure of biological age acceleration would show any difference between healthy subjects and those with certain known diseases. Table 9 shows the results grouped for subjects having chronic diseases such as diabetes, cardio vascular disease (CVD), and kidney disease. On average Δ_*g*_ = *BA*_*g*_−*BA* is lower for the individuals having chronic diseases (diabetes = −5.18, kidney = −3.66, and CVD = −2.92) whereas for all subjects Δ_*g*_ = −0.67. Those that do not suffer from any chronic disease have a Δ_*g*_ = 0.25 on average. We observe a similar pattern using ${\eta }_{g}=\frac{B{A}_{g}-BA}{CA}$ for the same partition. Positive and Negative refer to average of the subjects having positive and negative Δ respectively. Positive Δ and *η* corresponds to lower biological age than the chronological age (more healthy), while negative values correspond to higher biological age than the original age. % of negative Δ is higher for subjects with disease (74.53%, 65.38%, and 66.67%), compared with all subjects (56.25%). Subjects with no chronic disease have lowest proportion of negative Δs (52.25%).

**Table 9:** Performance of estimated biological age of subjects having different chronic diseases.

	Δ_*g*_					*η*_*g*_				
	Diabetes	Kidney	CVD	All-Subjects	Others	Diabetes	Kidney	CVD	All-Subjects	Others
Average	−5.18	−3.66	−2.92	−0.67	0.25	−0.10	−0.06	−0.05	0.00	0.02
Positive	7.49	9.20	7.89	8.84	8.98	0.17	0.20	0.18	0.21	0.22
Negative	−9.51	−10.47	−8.33	−8.07	−7.67	−0.19	−0.20	−0.17	−0.17	−0.16
% Pos	25.47	34.62	33.33	43.75	47.58	25.47	34.62	33.33	43.75	47.58
% Neg	74.53	65.38	66.67	56.25	52.42	74.53	65.38	66.67	56.25	52.42

These results show that the proposed ConvLSTM* estimated BA locomotor activity data can indeed capture significant information about the health status of the subjects.

### Comparison

Pyrkov *et al*.^7^ proposed a deep learning architecture for analyzing the physical activity data that is based on a one dimensional convolutional neural network (CNN) architecture. We also implemented a deep neural network (DNN) to estimate biological age (Our own architecture and implementation; motivated from the architecture of Putin *et al*.^22^, and also a basic CNN fed into an LSTM model (CNN + LSTM)). These models (DNN and 1D CNN) are used as comparative results. The results on mortality modeling using the Cox model and KM curves have shown the performance of the proposed ConvLSTM* in comparison with DNN and Pyrkov *et al*.’s^7^ 1D CNN and CNN + LSTM. See Tables 3 and 4 and Fig. 6. The results showed that the proposed ConvLSTM* method generally outperformed the 1D CNN, the CNN + LSTM model, or the DNN. Another way to compare the methods is by considering the estimated chronological age from the methods. Since the deep learning methods were trained to minimize the mean square error between the estimated and the original chronological age, we can compare the methods based on their performance in CA estimation.

Table 10 shows the mean absolute error (MAE), root mean square (RMSE), correlation (CORR), and R-squared value(R-sq) for all the deep learning methods discussed. Results are reported for both training and test datasets. We observe that ConvLSTM* (*λ* = 1) on the original dataset has the lowest MAE (12.6), RMSE (15.74), R-sq of 0.85, and best correlation (*ρ* = 0.62). ConvLSTM* with *λ* = 0 and *λ* = 0.9 had similar performance (*ρ* = 0.55 for both, R-sq of 0.85 and 0.80, and MAE of 13.21 and 13.4 respectively). While 1D CNN^7^ has the best R-sq (0.93) followed by the DNN network (R-sq = 0.89), for MAE and correlation ConvLSTM* and CNN + LSTM model performed better. They also required fewer epochs (10 compared with 100 for DNN and 500 for 1D-CNN). We have also considered 7 × 24 matrix representation followed by using LSTMs for sequences of 60 as a variation of ConvLSTM* architecture. We observe MAE = 16.45, *ρ* = 0.32 for the test datasets using this variation of architecture.

**Table 10:** Results of the Deep learning Age Prediction methods.

		1D CNN^7^	DNN	ConvLSTM*			*CNN* + *LSTM*
		(*λ* = 0.9)	(*λ* = 0.9)	(*λ* = 1)	(*λ* = 0)	(*λ* = 0.9)	(*λ* = 0.9)
Test	MAE	15.49	15.92	12.6	13.21	13.4	13.58
	RMSE	18.81	18.38	15.74	16.81	16.74	16.45
	CORR	0.45	0.45	0.62	0.55	0.55	0.54
	R-sq	0.93	0.89	0.85	0.85	0.80	0.71
Train	MAE	12.88	17.08	6.51	10.17	5.63	12.56
	RMSE	18.81	18.38	8.58	12.95	7.46	16.45
	CORR	0.52	0.79	0.92	0.79	0.94	0.64
	R-sq	0.88	0.74	0.75	0.70	0.82	0.59
	epoch	500	100	10	10	10	10

The above discussion demonstrates the specific benefits of ConvLSTM* over other deep learning methods when applied to locomotor activity data, namely, improved mortality modeling (using Cox PH, *χ*^2^-distance from the log-rank test, and using KM curves) and improved CA prediction (MAE, RMSE, correlation). The improved performance of the proposed ConvLSTM* can be attributed to (1) the use of a data representation that exploits the temporal patterns in the locomotor activity data, and (2) the use of a special deep learning model that combines the power of both CNN and LSTM. As discussed briefly in the introduction, there are several approaches to age estimation, using different types of data. Here, given the significant differences in methodology and datatypes involved, it is difficult to provide a detailed comparison with other non-deep learning approaches, or those that used other types of data. In general, the deep learning approaches on locomotor activity data tended to result in higher MAE when compared with methods that used other datatypes, for instance brain MRI^25^, or DNA methylation profiles^34,46^. However, the correlation (and *R*^2^-values) are generally similar. Our results using the Cox PH also shows that the performance in modeling mortality is similar to other popular BA methods, such as using the KD method on blood biomarkers.

#### Does improved CA estimation really imply reduced performance in BA estimation?

All the methods described above use supervised learning that learns in the form of minimizing the difference between estimated biological age and the chronological age itself. This difference has been called biological age acceleration^11^ in the literature. Pyrkov *et al*.^7^ suggested that an improvement in CA estimation can affect the significance of BA acceleration for a particular test that may involve health risks. This also relates to the issue of “paradox of biomarkers” as described by Klemera & Doubal^12^, and Hochschild^47^. However, our results show that the proposed ConvLSTM* approach results in a better estimation for chronological age (lower MAE, higher correlation) in comparison with the other deep learning methods. We have also shown that ConvLSTM* on the transformed data (using *λ* = 0, 0.9) have better BA acceleration and better performance in modeling all-cause mortality using both the Cox PH model and KM curves than 1D CNN, DNN, and CNN + LSTM. The normalized biological age acceleration (*η*) using the estimated BA from ConvLSTM* on the transformed activity values (*λ* = 0, 0.9, 1) resulted in a better overall performance in capturing health risks, for instance, in modeling all-cause mortality, when compared with the other deep learning methods, namely, 1D CNN^7^, CNN + LSTM, and DNN. These results seem to suggest that improved CA estimation may not always lead to a deterioration in BA estimation. The issue might be in how the estimated BA is used for further analysis, rather than the accuracy of the initial chronological age estimation. This clearly warrants further investigation, for instance, studying approaches that can combine the results from the fitting-based models that minimize the mean square error (MSE) with recent approaches (e.g. Pyrkov *et al*.^7^, Liu *et al*.^17^) that have used proportional risk models for developing methods to estimate biological age, rather than just testing the performance of estimated biological age.

## Conclusion

In this work, we studied biological age estimation using human locomotor activity. We applied a deep learning based framework to estimate biological age using Convolutional Long Short-Term Memory. We established that convolutional LSTM can be used to exploit temporal patterns in human locomotor physical activity to estimate biological age. The paper used different measures to compare performance in age estimation, including the traditional methods, (namely, MAE, RMSE, and correlation). To evaluate performance in biological age (BA) estimation, we introduced two new approaches, namely, relation with known health indices (WHtR, and SBSI), and relation with disease status (CVD, diabetes, and kidney diseases), in addition to traditional mortality modeling using Cox PH, *χ*^2^-distance from the log-rank test, and KM curves. Considering all the different methods for quantifying the performance of the estimated BA, the ConvLSTM* has the overall best result.

We identify some limitations of this study. One potential problem is the lack of control for certain demographics, for instance, socio-economic status, ancestry, etc. The available dataset was for one week. Although this is a time series data and for each individual we have 10080 (7 × 24 × 60) minutes of information, more data may reveal other important information not apparent from one week data. The device (ActiGraph AM-7164 piezoelectric accelerometer) that is used to collect the data was worn on hip by each individual. Sometimes the device was removed from the body (e.g. during shower). We believe, a water resistant smart watch or a wristband type device would be easier to use from a user perspective and hence the activity records would be more accurate.

## Supplementary information

Supplementary Materials of ”Deep Learning using Convolutional LSTM estimates Biological Age from Physical Activity”
