# Supplementary material for: Deep Learning using Convolutional LSTM estimates Biological Age from Physical Activity
Source: Sci Rep. 2019 Aug 6;9:11425. doi: 10.1038/s41598-019-46850-0 (PMC6684608; doi:10.1038/s41598-019-46850-0)
Supplement: Supplementary file 1 — Supplementary Materials of ”Deep Learning using Convolutional LSTM estimates Biological Age from Physical Activity” [file 41598_2019_46850_MOESM1_ESM.pdf]

Supplementary Materials of ”Deep Learning using Convolutional LSTM estimates Biological Age from Physical Activity”

[1] Syed Ashiqur Rahman, [2*] Donald A. Adjeroh

	$\lambda = 0$				$\lambda = 0.9$			
**N**	**SVM**		**RF**		**SVM**		**RF**	
	mae	correlation	mae	correlation	mae	correlation	mae	correlation
1	13.73	0.51	14.29	0.52	13.72	0.51	14.26	0.53
2	13.7	0.51	14.19	0.51	13.95	0.5	13.9	0.54
4	13.67	0.52	14.03	0.53	14.19	0.5	13.95	0.55
8	13.59	0.52	13.93	0.54	13.99	0.51	13.5	0.58
10	13.58	0.52	13.94	0.54	13.96	0.51	13.42	0.59
16	13.56	0.53	13.98	0.54	13.89	0.52	13.25	0.59
20	13.53	0.53	13.73	0.56	13.83	0.52	13.29	0.59
25	13.54	0.53	13.7	0.56	13.77	0.53	13.12	0.6
30	13.56	0.53	13.53	0.57	13.77	0.53	13.12	0.6
35	13.58	0.53	13.66	0.56	13.72	0.53	12.86	0.62
40	13.6	0.53	13.56	0.57	13.69	0.53	12.86	0.62

Table S1. Variations of window size for computing the moving averages after log-transformation.

	**SVM**		**RF**	
λ	mae	correlation	mae	correlation
0.2	13.50	0.53	13.42	0.58
0.3	13.45	0.54	13.42	0.58
0.4	13.46	0.54	13.33	0.59
0.5	13.48	0.54	13.23	0.6
0.65	13.53	0.54	13.03	0.61
0.75	13.57	0.54	12.99	0.61
0.85	13.65	0.53	13.01	0.61
0.9	13.72	0.53	12.86	0.62
0.95	13.74	0.53	12.98	0.61
1.1	13.86	0.53	12.87	0.61

Table S2. Choosing λ parameters for the Box-Cox transformation.

	Chi-Sq	p-value
1D CNN	42.98	2.49E-09
DNN	36.96	4.70E-08
CNN+LSTM ($\lambda = 0.9$)	29.08	2.16E-06
ConvLSTM* ($\lambda = 1$)	27.67	4.27E-06
ConvLSTM* ($\lambda = 0$)	23.98	2.52E-05
ConvLSTM* ($\lambda = 0.9$)	28.72	2.57E-06

Table S3. Results of the log-rank test applied on the gender and age cohort based normalized biological age acceleration group η using the estimated biological ages.

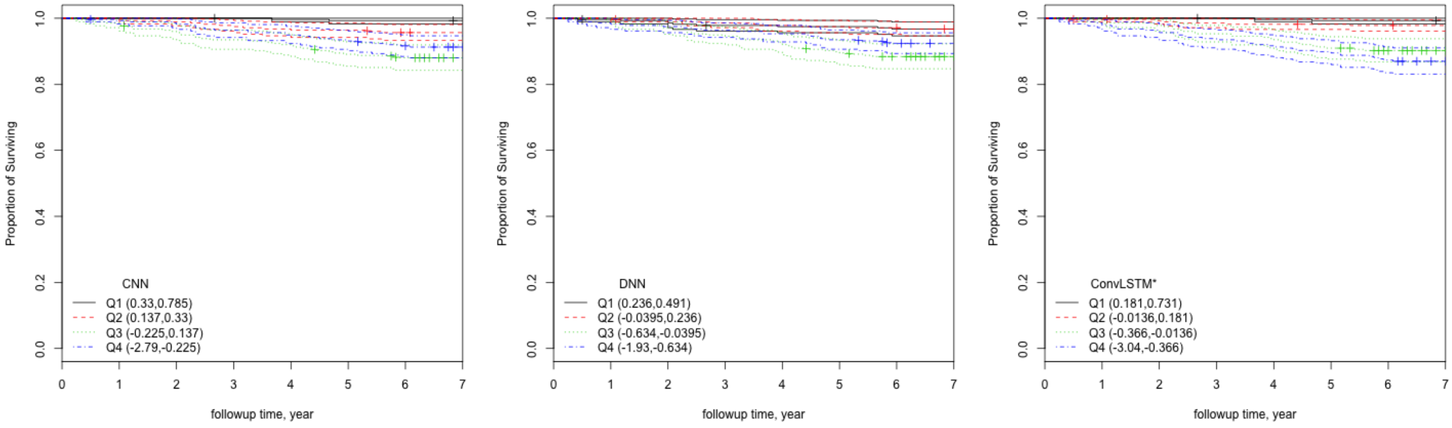

(a) $1DCNN_{BA}$ **(b)** DNN_{BA} **(c)** $ConvLSTM*_{BA}$

Figure S1. The Kaplan Meier curves with confidence intervals for estimated biological ages (BA) based on the physical activity applying $\eta = \frac{CA-BA}{CA}$ estimated biological ages- (a) 1D CNN (b) DNN, and (c) ConvLSTM*. Q1, Q2, Q3, and Q4 denote 1st, 2nd, 3rd, and 4th quartiles, respectively. The number of individuals in each Q is 276.

	PA Avg	Age	η	η_g
C-reactive protein	-0.08	0.05	0.00	-0.05
Glycated hemoglobin	-0.09	0.34	0.09	-0.19
Serum Albumin	0.17	-0.09	-0.01	0.10
Total Cholesterol	-0.03	0.18	0.11	-0.22
Serum Urea Nitrogen	-0.06	0.46	0.12	-0.22
Serum Alkaline Phosphatase	-0.07	0.06	-0.01	-0.04
Systolic blood pressure	-0.11	0.53	0.11	-0.31
Diastolic blood pressure	0.05	0.10	0.07	-0.14
Pulse	-0.08	-0.21	-0.08	0.09
High density lipoprotein	-0.02	-0.01	0.03	-0.07
Hemoglobin	0.15	-0.02	-0.01	0.07
Lymphocyte percent	0.06	-0.07	0.01	0.03
White blood cell count	-0.07	-0.05	-0.04	0.02
Hematocrit	0.15	-0.01	-0.01	0.07
Red blood cell count	0.15	-0.13	-0.04	0.15
Platelet count	-0.04	-0.12	-0.01	0.01

Table S4. Correlation between average physical activity, chronological age, η, η_g, and the biomarkers.

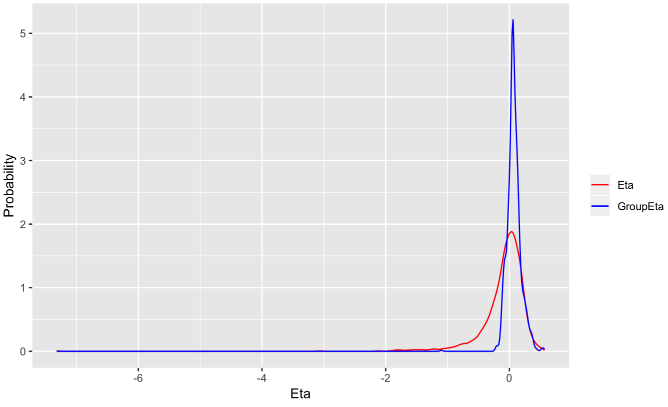

(a) ≤ 30

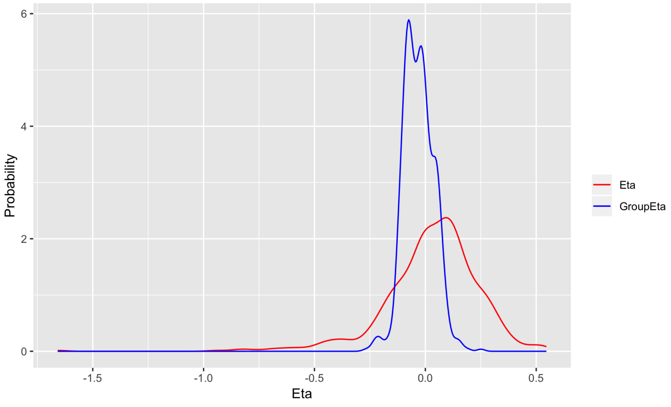

(b) $30-40$

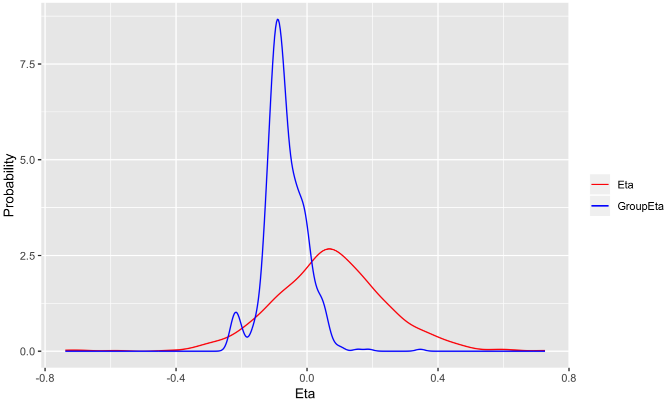

(c) $40-50$

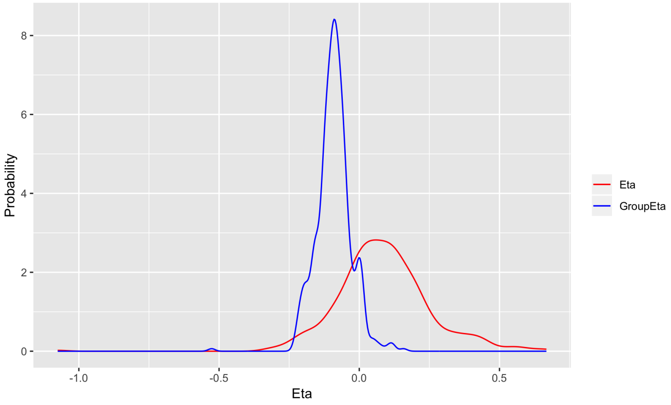

(d) $50-60$

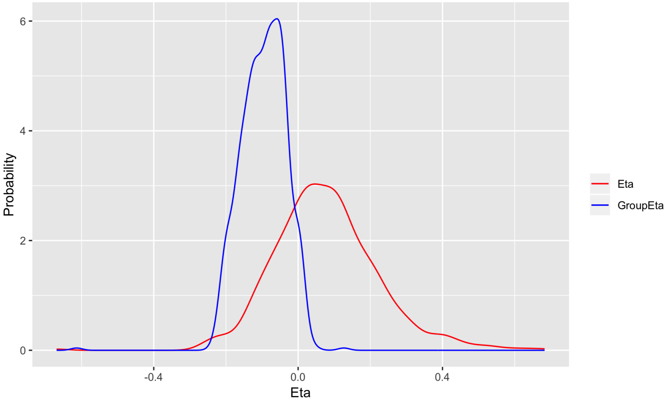

(e) $60-70$

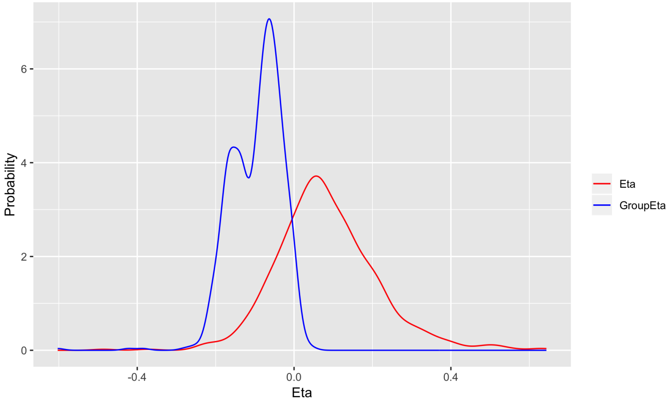

(f) > 70

Figure S2. Distribution of different variations of age acceleration (namely η, η_g) across age groups. We have grouped the subjects in different age categories ($\leq 30, 30-40, 40-50, 50-60, 60-70, 70+$).

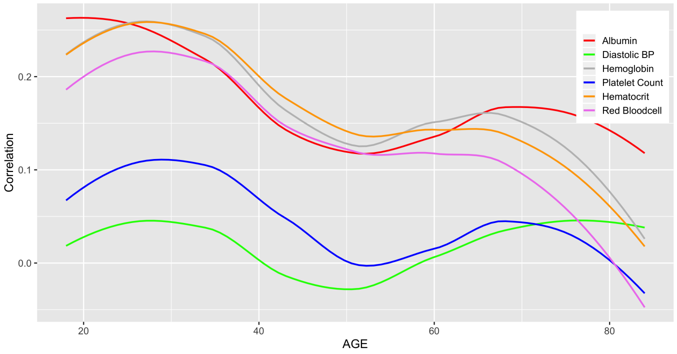

(a) Positively Correlated

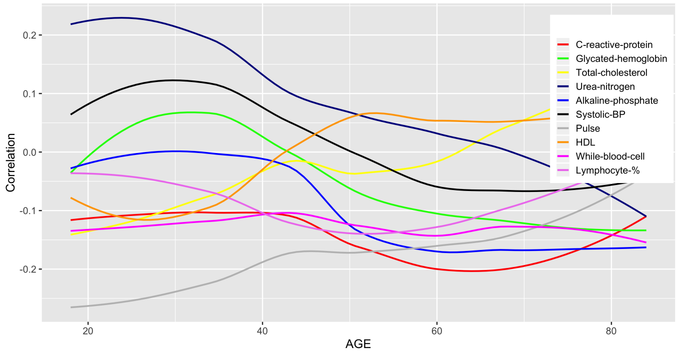

(b) Negatively Correlated

Figure S3. Correlation between average physical activity over age group with biomarkers. (a) biomarkers with predominantly positive correlation; (b) biomarkers with predominantly negative correlation.
